# Neurological Consequences of COVID-19: A Curious Case of Delayed Onset Guillain-Barre

**DOI:** 10.7759/cureus.25325

**Published:** 2022-05-25

**Authors:** Aditya K Devarakonda, Tanner R Stumpe, Ashley N Saucier, Thaddeus Riley

**Affiliations:** 1 Family Medicine, Augusta University Medical College of Georgia, Augusta, USA

**Keywords:** sars-cov-2, neurological sequelae, family medicine, covid-19, guillain-barre syndrome

## Abstract

SARS-CoV-2 is responsible for causing the COVID-19 pandemic and over 4 million deaths globally. Clinical symptoms range from asymptomatic infection, viral syndrome, and pneumonia, to acute respiratory distress syndrome. Guillain-Barre syndrome (GBS), an acute demyelinating inflammatory polyneuropathy, may be a manifestation of infection and must be recognized quickly by clinicians to avoid neurological deterioration in these patients. Here, we present an interesting case of GBS in a patient with a previous COVID-19 infection.

A 63-year-old male with a past medical history of hypertension, chronic obstructive pulmonary disease, obesity, and recent COVID-19 infection just five weeks prior to the presentation without COVID-19 vaccination presented to a family medicine clinic due to a history of falls as well as lower extremity numbness, weakness, and paresthesias for the past 36 hours. The patient’s MRI and lumbar puncture were unremarkable and the patient was transferred to a tertiary care center. The patient was diagnosed with GBS secondary to his COVID-19 infection five weeks prior. He received a standard five-dose regimen of 400 mg/kg/day of intravenous immunoglobulin and demonstrated rapid improvement in response to therapy.

Temporal factors associated with disease such as the seemingly delayed onset of symptoms after COVID-19 viral infection in comparison to other cases of GBS, as well as the rapid progression of symptomatology, are of note. Healthcare providers should still consider GBS as a possibility in patients with a relatively distant history of COVID-19 infections. Rapid progression of symptoms should also be monitored as this may result in earlier respiratory morbidity and mortality in the absence of appropriate diagnosis and treatment.

## Introduction

The primary mechanism of COVID-19 infection involves functional binding to the angiotensin-converting enzyme 2 (ACE2), which is highly expressed in the oral and nasal mucosa, nasopharynx, lungs, stomach, small intestine, colon, skin, lymph nodes, thymus, bone marrow, spleen, liver, kidney, and brain [1,2].

Symptoms of infection largely depend on the age, comorbidities, and the status of the immune system in the infected individual. In a meta-analysis performed by Alimohamadi et al., the most common presenting symptoms include fever (81%), cough (58.5%), fatigue (38.5%), dyspnea (26.1%), and presence of sputum (25.8%) [3].

In addition to the common clinical manifestations of SARS-CoV-2 infection, there is evidence that the infection can result in neurological sequelae. One such complication is Guillain-Barre syndrome (GBS), an acute demyelinating inflammatory polyneuropathy in which the immune system destroys peripheral nerves and nerve roots [3].

The clinical presentation of GBS includes decreased or absent deep tendon reflexes with characteristic findings of albuminocytologic dissociation on cerebrospinal fluid (CSF) analysis and decreased conduction neuromuscular nerve conduction studies (4). In this report, we present a case of COVID-19 associated with delayed onset development of GBS.

## Case presentation

A 63-year-old male with a past medical history significant for hypertension, chronic obstructive pulmonary disease, obesity, and past COVID-19 infection without COVID-19 vaccination presented to his primary care clinic due to falls. For the past 36 hours, he had been experiencing progressive lower extremity numbness, weakness, and paresthesias, accompanied by constipation and urinary retention. His physical exam was notable for decreased sensation of the umbilicus. The patient was afebrile and normotensive. His cardiac exam showed only mild tachycardia and lung sounds were present bilaterally with mild tachypnea. Muscle strength in the lower extremities was 0/5 and deep tendon reflexes were absent. Due to the progressive nature of the patient’s symptoms and concern for Guillan-Barre, the patient was transferred to the local hospital where his magnetic resonance imaging and lumbar puncture were unremarkable. The patient was then transferred to a tertiary care center for further management.

Three days after transfer to the tertiary care center, the patient developed increased work of breathing with a respiratory rate of 25, and O_2_ saturation of 93% of 5L nasal cannula, and blood pressure of 163/107 mmHg. Laboratory workup is significant for decreased arterial partial pressure of oxygen, the presence of red blood cells in the urine, elevated C-reactive protein and decreased sodium (Table 1).

**Table 1:** Patient laboratory results *CRP: C-reactive protein, *WBC: White Blood Cells, *RBC: Red Blood Cells, *PaO_2_: Partial Pressure of Arterial O_2_, *PaCO_2_: Partial Pressure of Arterial CO_2_

	Laboratory Test	Patient Results	Reference Range
Arterial Blood Gas	PaO_2_	68 mmHg	75–100 mm Hg
	PaCO_2_	29 mmHg	38–42 mm Hg
	pH	7.4	7.38–7.44
CMP	Serum Glucose	147 mg/dL	70–99 mg/dL (fasting)
	Sodium	130 mEq/L	136–145 mEq/L
	Potassium	4.0 mEq/L	3.5–5.0 mEq/L
	Bicarbonate	15 mEq/L	98–106 mEq/L
	Blood Urea Nitrogen	21 mg/dL	8–20 mg/dL
	Creatinine	1.00 mg/dL	0.70–1.30 mg/dL
	Alanine Aminotransferase	18 IU/L	10–40 U/L
	Aspartate Aminotransferase	31 IU/L	10–40 U/L
CBC	White Blood Cells	10,800 cells per microliter	4,000–11,000/μL
	Neutrophils	80.9%	50%–70%
	Lymphocytes	8.5%	30%–45%
	Hemoglobin	13.3 g/dL	14–18 g/dL
	CRP	1.30 mg/dL	≤0.8 mg/dL
Urinalysis	Blood	2+	0
	WBC	8 per high power field	0–5 cells/μL
	RBC	145 per high power field	0 cells/µL

The patient was intubated due to respiratory failure with an Erasmus GBS respiratory insufficiency score of 5 and received a full neurological evaluation along with an electromyogram (EMG) study. EMG results were consistent with demyelinating sensory and motor polyneuropathy. The patient had no detectable spontaneous activity of the left tibialis anterior or left abductor muscles of the leg. The patient’s left median and left ulnar nerves EMG showed prolonged latency with low amplitude, slow velocity, and absent F-waves.

He was diagnosed with GBS thought to be secondary to his COVID-19 infection that was diagnosed with five weeks prior. He received a standard five dose regimen of 400 mg/kg/day of intravenous immunoglobulin (IVIG). The patient demonstrated rapid improvement in response to therapy with significantly improved strength in his upper extremities on day 5 of IVIG therapy. He was extubated five days after initial intubation. The patient’s hospital course was complicated by small bowel obstruction which respond well to conservative therapy. The patient was discharged home three days after extubation.

## Discussion

Neurological damage from SARS-CoV-2 is multifaceted, including mechanisms such as direct damage to specific receptors, cytokine-related injury, secondary hypoxia, and retrograde travel along nerve fibers [4]. On initial presentation, this patient presented with loss of lower extremity motor function, autonomic dysfunction of the intestinal and genitourinary tract, signs of lower extremity polyneuropathy, and progressive ascending paralysis, which increased our suspicion of GBS.

Rapid diagnosis and treatment of GBS are essential in improving patient outcomes. The Brighton criteria for GBS consist of the following findings: bilateral and flaccid weakness of the limbs, decreased or absent deep tendon reflexes in weak limbs, monophasic illness pattern interval between onset and nadir of weakness between 12 hours and 28 days, subsequent clinical plateau, electrophysiologic findings consistent with GBS, albuminocytologic dissociation and the absence of an identified alternative diagnosis for weakness [5].

Our patient met five of seven of these criteria which would place him in the level 3 category for diagnostic certainty. He also had a rapid improvement in his condition with the standard five-dose IVIG therapy further supporting GBS as the likely mechanism of his acute symptoms. This patient’s COVID-19 infection occurred approximately five weeks prior to the onset of symptoms contrasts with other reports of post-COVID-19 GBS reporting times typically less than three weeks [6-8]. This patient also presented with a rapid onset and progression of symptoms with bowel and bladder involvement occurring within 36 hours. To our knowledge, there are no other cases that present with such rapid progression of symptoms.

The differences in temporal factors associated with disease such as the delayed onset of symptoms after viral infection in comparison to other cases as well as the rapid progression of symptomatology are of note. Healthcare providers should still consider post-viral GBS as a possibility in patients with a relatively distant history of COVID-19 infections. Rapid progression of symptoms should also be monitored as this may result in earlier respiratory morbidity and mortality in the absence of appropriate diagnosis and treatment.

## Conclusions

GBS is a severe sequela after a viral illness that can lead to rapid deterioration and eventual death if not diagnosed and treated quickly. With the high prevalence of COVID-19 and numerous instances of associated development of GBS, clinicians should remain vigilant. Even in cases of an atypical temporal presentation, clinicians who are treating patients with past COVID-19 infection and neurological symptoms commonly associated with GBS should ensure that patients are either definitively diagnosed with GBS or it is sufficiently excluded as a possibility.
